# Addition of intramuscular to vaginal progesterone for luteal phase support in fresh embryo transfer cycles: A cross-sectional study

**DOI:** 10.18502/ijrm.v20i9.12064

**Published:** 2022-10-10

**Authors:** Samaneh Jalaliani, Robab Davar, Farzad Akbarzadeh, Fatemeh Emami, Maryam Eftekhar

**Affiliations:** ^1^Research and Clinical Center for Infertility, Yazd Reproductive Sciences Institute, Shahid Sadoughi University of Medical Sciences, Yazd, Iran.; ^2^Department of Psychiatry, Psychiatry and Behavioral Sciences Research Center, Mashhad University of Medical Sciences, Mashhad, Iran.

**Keywords:** Luteal phase, Progesterone, Assisted reproductive technology, Embryo transfer.

## Abstract

**Background:**

Luteal phase deficiency is common in assisted reproductive technology and is characterized by inadequate progesterone production. Various studies have shown that administration of progesterone in fresh embryo transfer cycles increases the rate of clinical pregnancy and live birth rate. Progesterone administration has variable types: oral, vaginal, oil-based intramuscular, and subcutaneous.

**Objective:**

This study aims to compare the effect of adding intramuscular progesterone to the vaginal progesterone for luteal phase support in the fresh embryo transfer cycle.

**Materials and Methods:**

This study reviewed the information related to 355 women who had a fresh embryo transfer between March 2020 and February 2021 at the Yazd Reproductive Sciences Institute, Yazd, Iran. The participants population were divided into 2 groups based on the type of luteal phase support regime: group I (n = 173) received 400 mg vaginal progesterone alone twice a day from the day of ovum pick up; and group II (n = 182) received 50 mg IM of progesterone in addition to vaginal progesterone 400 mg twice a day from the day of ovum pick up. Chemical and clinical pregnancy rates were compared between groups.

**Results:**

The basic characteristics of groups were statistically similar. The rates of chemical and clinical pregnancy were higher in the vaginal plus IM progesterone group than in the vaginal progesterone group. Moreover, chemical pregnancy showed a significant difference between the groups (p = 0.011).

**Conclusion:**

Our findings demonstrated that the addition of IM progesterone to the vaginal progesterone improves the chemical pregnancy rate in fresh embryo transfer.

## 1. Introduction

The luteal phase starts after ovulation, supported by progesterone which increases implantation and the pregnancy rate in assisted reproductive technology (ART) cycles (1). Luteal phase deficiency is a common result of ART and is characterized by inadequate progesterone production, so luteal phase support (LPS) is needed for better implantation in the ART cycle (2). Progesterone supplementation is imperative to maintain implantation and early pregnancy until the luteo-placental shift, which occurs during the second trimester of pregnancy (3). Various studies have shown that supporting the luteal phase by administration of progesterone in fresh embryo transfer cycles increases the rate of clinical pregnancy and live birth rate (4-6). Progestogen can begin on the day of oocyte retrieval. or one day later, or the day of embryo transfer, and should continue until positive pregnancy test or 10-12 wk after gestation or until a negative serum human chorionic gonadotropin (HCG) (7).

The progesterone administration has variable types: oral, vaginal, oil-based intramuscular (IM), and subcutaneous progesterone (8). Vaginal and IM progesterone are preferred while oral progesterone alone is usually avoided because it is associated with inadequate bioavailability (9).

Some studies have shown that the use of vaginal progesterone causes a lower rate of miscarriage than IM progesterone (5, 10). The same percentage of pregnancies and miscarriages has been reported in participants receiving vaginal or IM progesterone (11). Therefore, there is an ongoing requirement to assess the LPS in fresh in vitro fertilization cycles (12).

However, there is a general agreement on the use of progesterone in fresh cycles; the choice of preparation, and its duration remains a matter of debate. So far, this study aimed to evaluate the effect of adding IM progesterone to vaginal progesterone on increasing pregnancy rate, and whether it reduces miscarriage in fresh embryo transfer cycles. The study also compared the results with those obtained from vaginal progesterone administration alone.

## 2. Materials and Methods

### Study population

This analytical cross-sectional study reviewed the medical records of infertile women who had a fresh embryo transfer between March 2020 and February 2021 at the Yazd Reproductive Sciences Institute, Yazd, Iran. 448 fresh embryo transfer cycles were reviewed. Women with incomplete data were removed from the study.

The inclusion criteria were infertile women aged between 18-40 yr and candidates for the antagonist protocol and fresh embryo transfer. On the other hand, the women candidates for frozen embryo transfer; those with uterine malformation or adhesions, severe adenomyosis or endometriosis, severe male factor, severe maternal systemic disease, and candidates for preimplantation genetic testing were excluded from the study. A total of 355 participants met the inclusion criteria of the study.

### Study protocol

Women were stimulated by gonadotropin from the 2 ${}^{\text{nd}}$ day of cycle. The initial gonadotropin dose ranged from 150-300 IU per day. Follicular monitoring was done by vaginal sonography from the 6 ${}^{\text{th}}$ day of stimulation. Gonadotropin dose was adjusted according to the ovarian response.

With follicular diameter ≥ 14 mm, gonadotropin-releasing hormone (GnRH)-antagonist 0.25 mg was administered daily and continued until the day of triggering. When at least three follicles reached a mean diameter of 17 mm, 5,000-10,000 IU of HCG or dual triggering with hCG plus GnRH agonist was done. Oocyte retrieval was performed 34-36 hr after triggering. Embryo transfer was done on day 2 or 3 after oocyte retrieval.

The study population were divided into 2 groups based on the LPS regime: Group I received 400 mg of vaginal progesterone alone twice a day from the day of ovum pick up, and group II received 50 mg IM progesterone daily in addition to vaginal progesterone 400 mg twice a day from the day of ovum pick up. Chemical pregnancy was defined as serum beta hCG ≥ 50 IU/L, 14 days after embryo transfer. Clinical pregnancy was defined as presence of fetal heart activity in ultrasonography done 4 wk after embryo transfer. LPS was continued until 12 wk of gestation.

### Data collection

Demographic characteristics, including age, duration and type of infertility, and body mass index, as well as laboratory information, including anti-mullerian hormone (AMH), endometrial thickness, embryo grading, and type of progesterone consumption were recorded for all women. Furthermore, the rates of positive or negative chemical and clinical pregnancy were recorded in this study.

### Ethical considerations 

The study protocol was reviewed and approved by the Ethics Committees of Yazd Reproductive Sciences Institute, Yazd, Iran (Code: IR.SSU.RSI.REC.1399.040). The data were coded and then recorded into the checklists to maintain data confidentiality.

### Statistical analysis

Descriptive data were summarized as mean ± SD and/or percentage. The normality of the data was checked before the analysis by the one-sample Kolmogorov-Smirnov test. Moreover, Chi-square test was used to determine the relationship between progesterone intake and the pregnancy rate. The Independent-Sample *t* test was used to examine the effects of AMH, Age, and body mass index. The collected data were analyzed with Statistical Package for the Social Sciences, version 25.0, SPSS Inc, Chicago, Illinois, USA (SPSS). A p-value < 0.05 was considered statistically significant.

## 3. Results 

This study was conducted on 355 women who received vaginal progesterone (n = 173; 48.7%) and vaginal progesterone along with IM progesterone (n = 182; 51.3%). The women's median age, body mass index, and median duration of infertility were the same between groups. Table I presents the general and demographic characteristics of the groups.

The infertility etiologies between groups was similar table II the mean age of the women was 34.11 ± 5.46 yr (age range: 18-40 yr). No statistically significant difference was observed between groups (Table I). The mean of anti-mullerian hormone (p = 0.315), infertility duration (p = 0.582), embryo grading (p = 0.376), and embryo number (p = 0.061) was the same between groups. The most frequent embryo grading were B (46.8%) and A (30.7%), respectively.

Chemical (37.4%) and clinical (23.6%) pregnancy rates were higher in the vaginal progesterone along with IM progesterone group, compared to the vaginal progesterone group (Table III). The statistical analysis showed that the difference was significant (p = 0.011) for chemical pregnancy; however, it was not significant in the clinical pregnancy (p = 0.080).

**Table 1:** General and demographic characteristics of the groups


**Variables**	**Vaginal progesterone** **group (n = 173)**	**Vaginal progesterone + IM progesterone (n = 182)**	**P-value**
**Age (yr)**	33.55 ± 4.40	32.72 ± 4.53	0.080 ${}^{*}$
**BMI (kg/m²)**	26.41 ± 3.92	26.19 ± 4.47	0.618 ${}^{*}$
**ET (mm)**	9.09 ± 1.37	9.48 ± 1.59	0.076 ${}^{*}$
**AMH (ng/ml) **	3.17 ± 2.48	3.47 ± 2.73	0.31 ${}^{**}$
**Infertility duration (yr) **	6.41 ± 3.63	6.27 ± 3.76	0.58 ${}^{**}$
Data are presented as Mean ± SD. *Student *t* test, **Mann-Whitney test, BMI: Body mass index, ET: Endometrial thickness, AMH: Anti-Mullerian hormone, IM: Intramuscular			

**Table 2:** Comparison of the infertility etiologies between groups


**Infertility etiologies**	**Vaginal progesterone group**	**Vaginal progesterone + IM progesterone**	**P-value**
**PCOS**	32 (18.5)	41 (22.5)	
**DOR**	40 (23.1)	34 (18.7)	
**Male factor**	30 (17.3)	32 (17.6)	
**Tubal factor**	5 (2.9)	4 (2.2)	
**Endometriosis**	5 (2.9)	4 (2.2)	
**Unexplained**	37 (21.4)	46 (25.3)	
**Mixed**	24 (13.9)	20 (11.0)	0.782
Data presented as n (%), Chi-square tests, PCOS: Polycystic ovary syndrome, DOR: Diminished ovarian reserve, IM: Intramuscular			

**Table 3:** Comparison of ART outcomes between groups


**Variables**		**Vaginal progesterone group**	**Vaginal progesterone + IM progesterone**	P-value*
**Fetal grade**				
	**A**	48 (27.7)	61 (33.5)	
	**B**	81 (46.8)	85 (46.7)	
	**C**	44 (25.5)	36 (19.8)	0.33
**Total embryo transfer**				
	**1**	50 (28.9)	37 (20.3)		
	**2**	123 (71.1)	145 (79.7)	0.061
**Chemical pregnancy**		43 (24.9)	68 (37.4)		0.011
**Clinical pregnancy**		28 (16.2)	43 (23.6)	0.080
Data presented as n (%), *Chi-Square test, ART: Assisted reproductive technology, IM: Intramuscular				

## 4. Discussion 

The prescription of vaginal progesterone as an effective drug for luteal support has been well recognized in many studies (8, 13, 14). However, despite the common use of the progesterone for luteal support, the best route and dosing of progesterone is still unidentified (15). This study evaluated the effect of IM progesterone along with vaginal progesterone on in vitro fertilization cycle outcomes in fresh embryo transfer. This results showed that the distribution of AMH, infertility duration, fetal grade, and embryo number were the same between groups. Moreover, the rates of chemical and clinical pregnancy were higher in the vaginal progesterone and the IM progesterone group, compared to the group that received vaginal progesterone alone. However, chemical pregnancy showed a significant difference between groups. In a normal menstrual cycle after mid-cycle luteinizing hormone (LH) surge and monofollicular ovulation, peripheral progesterone concentration increased. “It is necessary for the secretory transformation of the endometrium, successful implantation and maintenance of early pregnancy. Insufficient progesterone secretion at the time of implantation may cause early pregnancy loss or lack of implantation" (16).

In the ovarian stimulation cycle, downregulation and pituitary desensitization with GnRH analogs results in the reduced endogenous release of gonadotropins in the early luteal phase. Furthermore, supraphysiological concentrations of estradiol and progesterone following ovarian stimulation and multiple corpus luteums have negative feedback on the hypothalamus and reduce the amount of LH released from the pituitary (17).

In fresh embryo transfer cycles, multiple corpora luteums are accessible in both ovaries. However, there is a relative mid-luteal phase hCG/LH deficiency after the aspiration of granulosa cells during oocyte retrieval (18). Exogenous progesterone is usually administered for LPS in the ovarian stimulation cycle and fresh embryo transfer (19). LPS via progesterone in fresh and frozen embryo transfer cycles increases pregnancy (5). LPS in ovarian stimulation cycles is required due to the iatrogenic effects of exogenous hormones on suppressing the secretion of endogenous gonadotropins (20).

A review article demonstrated that the oral, vaginal, subcutaneous, and IM use of progestrone is beneficial for clinical pregnancy rates and progesterone supplementation is considered mandatory for LPS in the ART cycle (21). IM progesterone results in higher concentration and more sustained serum levels than vaginal route; however, vaginal regimens achieve higher endometrial concentrations. It has been suggested that these higher local progesterone concentrations may not provide optimal support for better pregnancy outcome and IM progesterone has been suggested for better luteal support and greater uterine quiescence (13).

A survey on 408 ART centers in 82 countries found that about 77% of cycles used vaginal progesterone alone to support the luteal phase (8). However, the vaginal progesterone has some disadvantages, such as vaginal irritation or discharge in some women (22). Rare side effects like acute eosinophilic pneumonia have been reported after IM progesterone supplementation (23, 24).

One study reported that the addition of the IM progesterone to vaginal progesterone to support the luteal phase in fresh embryo transfer cycles increases pregnancy rate (10). Furthermore, the combined use of IM and vaginal progesterone in comparison to vaginal progesterone only leads to a reduced abortion rate and increased pregnancy rate (25). This study showed that the rate of chemical and clinical pregnancy was higher in the vaginal progesterone and the IM progesterone group, compared to the vaginal progesterone group. However, chemical pregnancy showed a significant difference between groups.

Similar to our study, a Cochrane review indicated that the combination therapy had no statistically significant differences between clinical pregnancy and miscarriage (26).

### Limitations and suggestions 

One limitation of this research is its retrospective nature. Moreover, according to our criteria, most of the gynecologic disorders that could affect endometrial receptivity were excluded. So, these results cannot cover the women with insufficient endometrial receptivity. Future studies are recommended to be conducted on the efficacy of vaginal progesterone and IM progesterone during the early implantation period.

## 5. Conclusion 

This study attempted to show the effect of adding IM progesterone to vaginal progesterone for LPS on pregnancy rate in fresh embryo transfer cycles. The results showed that the rate of chemical and clinical pregnancy was higher in the vaginal progesterone and the IM progesterone group. Chemical pregnancy showed a significant difference between groups. In summary, the utilization of the IM progesterone and vaginal progesterone appears to have some benefits in terms of successful pregnancy. It is suggested to add IM progesterone to vaginal progesterone for LPS in routine protocols. Since available data are not strong enough, the efficacy of IM progesterone along with vaginal progesterone should be further investigated.

##  Conflict of Interest

The authors declare that there is no conflict of interest.
